# Misoprostol versus oxytocin for labor induction in term and post-term pregnancy: randomized controlled trial

**DOI:** 10.1590/S1516-31802003000300003

**Published:** 2003-05-01

**Authors:** Márcia Maria Auxiliadora de Aquino, José Guilherme Cecatti

**Keywords:** Misoprostol, Oxytocin, Prostaglandins, Labor induction, Randomized controlled trial, Misoprostol, Ocitocina, Prostaglandinas, Indução, Parto, Ensaio, Clínico, Controlado, Aleatorizado.

## Abstract

**CONTEXT:**

Misoprostol, a synthetic E_1_ methyl analog prostaglandin, is at present receiving attention as a cervical modifier and labor induction agent. However, there is still a need for better determination of its safety and effectiveness.

**OBJECTIVE:**

To compare intravaginal misoprostol versus intravenous oxytocin for cervical ripening and labor induction in pregnant women with unripe cervices.

**DESIGN:**

Randomized controlled trial.

**SETTING:**

The study was performed at the Leonor Mendes de Barros Maternity Hospital between November 1998 and December 2000.

**PARTICIPANTS:**

210 pregnant women with intact membranes and indication for labor induction were selected.

**PROCEDURES:**

The women randomly received 25 μg of vaginal misoprostol every 4 hours, not exceeding 8 doses (105 women), or oxytocin in a continuous infusion (105 women).

**MAIN MEASUREMENTS:**

The main parameters measured were: latent period, time from induction to vaginal delivery, delivery route, occurrence of vaginal delivery with time, occurrence of uterine tonus alterations, hypoxia and neonatal morbidity. To verify the statistical significance of the differences between the groups, the chi-squared, Student *t* and log-rank tests were used.

**RESULTS:**

There were no significant differences between the groups concerning conditions for labor induction, age, parity, race, marital status, family income, initial Bishop Index and number of prenatal visits. The cesarean section rate, latent period and period from induction to vaginal delivery were significantly lower for the misoprostol group. With regard to uterine tonus alterations, tachysystole was significantly more common in the misoprostol group. However, there was no difference in hypoxia and neonatal morbidity between the groups.

**CONCLUSION:**

25 μg of misoprostol used vaginally every 4 hours is safer and more efficient for cervical ripening and labor induction than oxytocin.

## RESUMO

**CONTEXTO:**

Na atualidade, misoprostol, um metilanálogo sintético da prostaglandina E1, vem recebendo atenção como um agente maturador do colo e indutor do trabalho de parto. No entanto, ainda é necessário determinar melhor sua segurança e eficácia.

**OBJETIVO:**

Comparar misoprostol intravaginal versus ocitocina intravenosa para amadurecimento cervical e indução de parto em mulheres com colo uterino desfavorável.

**LOCAL:**

Hospital e Maternidade Leonor Mendes de Barros, em São Paulo, SP, no período de novembro de 1998 a dezembro de 2000.

**PARTICIPANTES:**

210 gestantes com membranas íntegras e indicação para indução do parto.

**PROCEDIMENTOS:**

As mulheres, após a aleatorização, receberam misoprostol vaginal na dose de 25 μg a cada quatro horas, não excedendo oito doses (105 mulheres) ou ocitocina em uma infusão contínua (105 mulheres).

**VARIÁVEIS ESTUDADAS:**

Período de latência, tempo da indução ao parto vaginal tipo de parto, ocorrência de parto vaginal em função do tempo, ocorrência de alterações do tônus uterino, hipóxia e morbidade neonatal. Para verificar a significância estatística das diferenças entre os grupos, utilizaram-se os testes de qui-quadrado, *log-rank* e t de Student.

**RESULTADOS:**

Não houve diferença significativa entre os grupos referentes às indicações de indução, idade, paridade, raça, renda familiar, índice inicial de Bishop e número de consultas de pré-natal. A incidência de cesárea, o período de latência e o período da indução ao parto vaginal foram significativamente menores para o grupo de misoprostol. Das alterações do tônus uterino, a taquissistolia foi significativamente mais comum no grupo do misoprostol, entretanto hipóxia e morbidade neonatal não foram diferentes entre os grupos.

**CONCLUSÃO:**

25 μg de misoprostol vaginal usados a cada quatro horas é seguro e mais eficiente para o amadurecimento cervical e indução do trabalho de parto do que a ocitocina.

## INTRODUCTION

There are many different situations in obstetrics where there is the need for labor induction in women with unripe cervices. This indication stems from a situation where the continuation of pregnancy may be life-threatening for the mother and/or fetus. Such induction is frequently prolonged, exhausting and very often unsuccessful, resulting in a cesarean section.^1-4^

Among the pharmacological agents used for labor induction, oxytocin and prostaglandins are the most common. Several studies have shown that continuous intravenous infusion of oxytocin is less efficient, particularly when there are unfavorable cervical conditions, leading frequently to a cesarean section, because of induction failure.^2,4,5^ In such cases, another pharmacological agent, perhaps a prostaglandin, should be used to favor cervical ripening, at least initially.

Several studies have been performed, especially in developed countries, using pros-taglandin E2 (dinoprostone) gel for cervical ripening and labor induction, particularly in cases where the Bishop Index^6^ is lower than seven, in which its effectiveness was shown. However, the widespread use of this drug is limited because of its high cost and thermal instability, which leads to difficult storage, plus the occasional need to use oxytocin after cervical ripening.^7,8^ In Brazil, this drug is not at present being marketed.

Misoprostol (Cytotec^®^), a synthetic E1 methyl analog prostaglandin, is at present receiving more attention as a cervical modifying agent and labor inductor, as it has the advantages of low cost, stability in relation to temperature, easy handling and storage, and also easy administration (vaginal or endocervical).^8-10^ However, there is still a need to better establish its safety so as to avoid hyperstimulation syndrome, which could result in undesirable consequences for the newborn.

During the last few years, many studies have been carried out on live fetus pregnancies, to establish the best dose, administration route and interval between doses for this cervical ripening and labor induction drug and compare it to oxytocin and dinoprostone.^8,11,13^ A meta-analysis performed in 1998 concluded that vaginal misoprostol seems to be more effective for cervical ripening and labor induction than the conventional methods. However, it emphasized the need for complementary studies in order to achieve safer results in relation to hyperstimulation syndrome and perinatal results.^14^

According to the published studies, the vaginal dose of misoprostol that has shown greatest safety and efficiency for the mother and newborn is 25 μg at 4-6 hourly intervals.^1,12,17^ In fact, a recent study on the pharmacokinetics of this drug showed that its serum levels, after vaginal administration, remain stable for at least 4 hours, suggesting that the ideal time interval between doses should be greater than 4 hours.^18^

The incidence of cesarean deliveries is quite high in Brazil. Some of them take place because of elective anticipation due to maternal and/or fetal complications that are associated with a high level of unsuccessful oxytocin induction.^19^ In view of this, a randomized controlled trial was proposed, in order to study the safety and effectiveness of vaginal misoprostol given at four-hourly intervals for cervical ripening and labor induction, in comparison with oxytocin infusion, in pregnant women with unripe cervices and live fetuses.

## METHODS

This was a randomized controlled clinical trial. The sample, 210 pregnant women requiring labor induction for any clinical and/or obstetric reason, was selected between November 1998 and December 2000. The inclusion criteria were: medical indication for labor induction; singleton gestation; gestation age greater than 36 weeks; vertex presentation, intact membranes; Bishop Index less than 6; no labor occurring; and normal fetal heart rate. The exclusion criteria were: pelvic dystocia; estimated fetal weight greater than 4,000 g or evidence of cephalopelvic disproportion; placenta previa or any unexplained vaginal bleeding; parity > 5; fetal malformation; previous uterine scar; any situation when vaginal delivery was not indicated; and any contraindication to the use of misoprostol.

Sample size was calculated on the basis of the results from a previous study using misoprostol and oxytocin with the same objective.^2^ Using an alpha error of 0.01 and a beta error of 0.05, the size of the sample was estimated as 105 women for each group.

After being interviewed, the women who met the eligibility criteria were invited to voluntarily participate in the study. Those who accepted were carefully informed of the aims and procedures of the study and then asked to sign the informed consent form. Following this, the fetal heart rate was monitored to ensure fetal wellbeing before the onset of induction. The random allocation procedure was determined by opening a sequentially numbered envelope, thereby determining whether that woman should receive either misoprostol or oxytocin. This random sequence was generated by computer and the investigator was unaware of the sequence.

For women receiving misoprostol, 25 μg were administered in the posterior fornix of the vagina. The dose was repeated every 4 hours, until a pattern of at least 3 contractions every 10 minutes was obtained. The maximum dose was 200 μg. If this contraction pattern had not been reached by four hours after the administration of the eighth dose, induction was considered to have failed. After the ideal pattern of contractions was reached, misoprostol was no longer administered.

For the women in the oxytocin group, an intravenous infusion of 2 mU/min was used, which was doubled at 30-minute intervals until the appropriate contraction pattern was obtained. The infusion dose was increased to a maximum of 20 mU/min, at which point it was then maintained constant. If the contraction pattern had not been induced by the time that 15 IU of oxytocin had been infused, the induction was also considered to have failed. Even after the ideal pattern was reached, oxytocin flow was continued.

As soon as the patients presented the desired contraction rate, monitoring of fetal heart rate and intrapartum uterine activity was performed. Amniotomy was carried out when the Bishop Index was greater than 7 and cervical dilation was greater than 6 cm.

In cases of tachysystole (6 or more contractions every 10 minutes) and uterine hypertonus/hypersystole (uterine contraction with a duration of 2 minutes or more), and in the absence of altered fetal heart rate monitoring, the reversion of such alterations was done by means of the usual procedures and, if necessary, tocolysis was used (terbutaline, 0.05 mg intravenously). In the cases where there was no reversion of the abovementioned alterations, or if hyperstimulation syndrome (tachysystole or uterine hypertonus/hypersystole with abnormal fetal heart rate) or fetal hypoxia (thick meconium and/or fetal monitoring alteration) were also present, induction was interrupted and a cesarean section was performed.

For data analysis, the comparability of the groups was initially evaluated through the distribution of control variables, in order to check the randomization process. To verify the statistical significance of the differences between the groups, the chi-squared and Student *t* tests were used respectively for categorial or continuous variables. To evaluate Bishop Index changes, the latent period (time from the beginning of induction to the onset of labor) and the time from induction to vaginal delivery, survival analysis was used. The statistical significance was evaluated via the Kaplan-Meyer method, through the log rank test. For the main dependent variable (delivery type) and variables related to safety, the risk ratio and their corresponding 95% confidence intervals (CI) were calculated. Finally, for delivery type alone, the number needed to treat (NNT) and its 95% CI was also estimated. The value of statistical significance adopted was 5%.

This study received approval from the Institutional Review Board of the participating institution.

## RESULTS

Out of the total of 210 women, 105 were randomly allocated to the use of misoprostol and 105 to oxytocin. In order to demonstrate the randomization process for the groups, the results from the control variables are presented. No significant differences between the groups concerning age, parity, race, marital status, family income, initial Bishop Index and number of prenatal visits were observed. The most common indication for labor induction was post-term pregnancy in both groups (Table 1).

**Table 1:** Characteristics of women and conditions indicating labor induction according to the assigned treatment (in %)

Characteristics	Misoprostol (105)	Oxytocin (105)	χ^2^	p value
Age < 25 years	58.1	52.4	0.48	0.49
White	68.6	75.2	0.85	0.36
Married	46.7	60.0	3.23	0.07
Family income < 4 mw*	52.4	59.0	0.03	0.87
Nulliparous	61.9	51.4	1.94	0.16
Up to 5 prenatal visits	24.8	38.1	2.27	0.13
Initial Bishop ≤ 3	76.2	82.8	1.05	0.30
Indication for labor induction			8.87	0.06
Hypertension	11.4	23.8		
Post-Term	61.9	60.0		
Oligohydramnios	0.9	0.3		
Diabetes	0.5	0.4		
Others	12.4	9.5		

* *minimum wage (R$180.00 per month).*

When the groups were analyzed in relation to the mean time between the beginning of induction and the onset of labor, there was a significant statistical difference. Labor (3 contractions per 10 minutes) started on average within 253 minutes in the misoprostol group, versus 352 minutes in the oxytocin group. Regarding the time interval from the start of induction to the occurrence of vaginal delivery, there was also a significant difference. The mean time for this interval was shorter for the misoprostol group (Table 2).

**Table 2:** Time interval for latent period and vaginal delivery occurrence according to the labor induction method

Time interval	Misoprostol	Oxytocin	p value
Latent period (minutes)	252.9 ± 149.1	352.3 ± 151.9	< 0.0001
N	104	92	
Time from induction to vaginal delivery (hours)	10.6 ± 4.4	14.8 ± 5.1	< 0.0001
N	85	67	

*All values are means ± standard deviation (SD).*

In the misoprostol group, 81% of women delivered vaginally within 24 hours from the start of induction and the same occurred for 62% in the oxytocin group. When induction success within the first 12 or 18 hours was evaluated, the percentage was significantly higher for the misoprostol group. Vaginal delivery occurred in 81% of the women in the misoprostol group versus 64% in the oxytocin group, which was also a significant difference (Table 3). Using misoprostol for cervical ripening and labor induction represented a 47% reduction in the risk of having a cesarean section (risk ratio = 0.53) and this also represented the fact that almost 6 cases would have to be treated with misoprostol instead of oxytocin in order to avoid one cesarean section (number needed to treat = 5.8).

**Table 3:** Type of delivery according to the drug used for induction

Type of delivery	Misoprostol		Oxytocin	
Vaginal (total)	85 (81%)		67 (64%)	
Vaginal within 12 h		65 (62%)		26 (25%)
Vaginal within 18 h		78 (74%)		45 (43%)
Vaginal within 24 h		85 (81%)		67 (64%)
Cesarean section	20 (19%)		38 (36%)	
**Total (n)**	**105**		**105**	

*χ^2^= 6.88; p = 0.008; risk ratio for cesarean section = 0.53; 95% confidence interval (0.33 – 0.84); number needed to treat = 5.8 (5.1 – 6.5).*

In the oxytocin group there were 13 cases of failed induction while in the misoprostol group there was only one. When prevalence of abnormal uterine contractions between the groups was analyzed, tachysystole was more prevalent in the misoprostol group, with statistical significance: there were 27 cases in the misoprostol group versus 9 in the oxytocin group. Prevalence of fetal hypoxia was higher in the misoprostol group. There were 14 cases in the misoprostol group versus 10 in the oxytocin group, presenting no statistical significance (Table 4).

**Table 4:** Occurrence of tachysystole, hyperstimulation syndrome and hypoxia, according to the drug used for labor induction

	Misoprostol	Oxytocin	Risk ratio	95% confidence interval
Tachysystole	27	09	3.00	1.49 – 6.05
Hyperstimulation syndrome	03	04	0.94	0.22 – 4.07
Hypoxia	14	10	1.19	0.82 – 1.72

**Figure 1:**
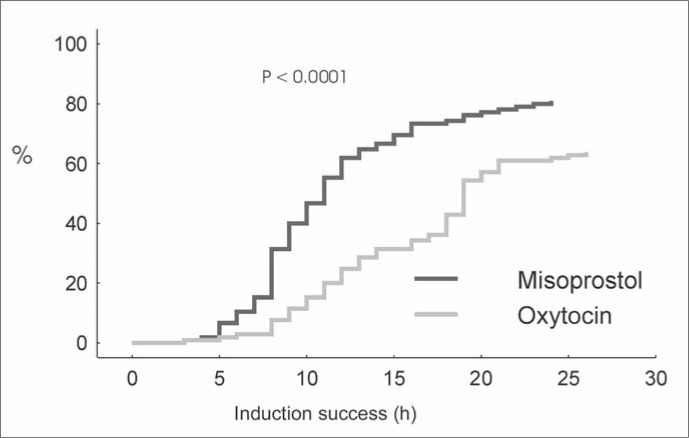
Distribution of the labor induction success rate, according to the method used.

Among all the newborns, there were 43 cases of neonatal morbidity (evaluated through their admission to the intensive care unit, number of days of hospitalization and respiratory alterations), 21 in the misoprostol group and 22 in the oxytocin group, which presented no significant difference. When Apgar Index < 7 at the fifth minute of life was analyzed, one case in the misoprostol group and 2 in the oxytocin group were found, again presenting no statistical significance. The survival analysis showed that the period from the beginning of induction until vaginal delivery was significantly shorter in the misoprostol group (Figure 1).

## DISCUSSION

This study showed that a vaginal 25 μg dose of misoprostol at four-hourly intervals might be a more effective agent than oxytocin for cervix ripening and labor induction, for term and post-term pregnant women with unripe cervices.

The present study showed that the average time interval to the onset of labor and also to the occurrence of vaginal delivery was significantly shorter in the misoprostol group. These same results have also been found by many other authors.^2,11^ Campos et al.^2^ studied misoprostol (50 μg, single dose, vaginally) and oxytocin (2 to 32 mU/min) and found an average of 552 min to the beginning of labor for the misoprostol group, versus 745 min for the oxytocin group, presenting statistical significance. These findings are consistent with our results.

An American study^3^ demonstrated that the average time interval until the occurrence of vaginal delivery was also shorter for misoprostol (50 μg at four-hourly intervals) than for oxytocin (11 hours versus 18), presenting statistical significance. In Turkey, another study showed the same result, presenting quite similar averages when compared to this study (9 hours versus 15).^11^

The average differences in these intervals are related to the dose, route and administration interval. There are also some studies on pregnant women with favorable cervices for induction or previous administration of dinoprostone before the start of induction.^3,11^

Some comparative studies between these two drugs have shown that the incidence of cesarean sections is higher in the oxytocin group,^2-5^ and this was also shown in our investigation: the rate was almost doubled for the oxytocin group. Other authors have found higher rates in the misoprostol group, giving statistical significance. Such occurrences might be explained by higher doses and/or shorter administration intervals, which may be associated with a higher incidence of abnormal uterine contractility and meconium.^1,2^

In our study, there were 14 cases of failed induction, 13 of them in the oxytocin group, showing a statistically significant difference between the groups. This was also found in the study by Kadanali et al.^11^

Our study showed no differences between the groups regarding Apgar index at the fifth minute of life and perinatal results. The majority of studies have shown that when peri-natal results are evaluated by means of Apgar score, cord pH, admission to intensive care unit, number of days of hospitalization, meconium passage syndrome or hyperbilirubinemia, there are no differences between the groups, which confirms the findings of the present study.^2,4,11^

In relation to uterine hypercontractility, only tachysystole was different between the groups: it was more prevalent in the misoprostol group. However, there is disagreement between studies concerning uterine hypercontractility. Some studies report greater incidence of uterine hypertonus and hyperstimulation syndrome.^1,4^

Again, the hypothesis is that the dose, interval and administration route are involved in this response. Moreover, it is inferred that the tachysystole occurring with the use of misoprostol does not involve alterations in fetal vitality, because uterine tonus does not change. This was shown in the study by Campos at al.,^2^ which monitored the intrauterine pressure in some women and found that the average intensity of the contractions and the uterine tonus did not differ between the two groups (misoprostol versus oxytocin).

The findings of alterations in uterine contractility and possible undesirable effects on the fetus have been leading researchers to perform more studies to check that the use of misoprostol does not necessarily imply the occurrence of hyperstimulation syndrome. Such a need was emphasized in the latest systematic update review published in the Cochrane Library.^14^

The first investigation done by Wing et al.^1^ was based on the same dose and administration interval as previously used by Sanchez-Ramos (1993).^3^ There was significantly higher incidence of tachysystole and meconium passage syndrome with misoprostol (50 μcg every 3 hours) than with dinoprostone. A second study was performed by Wing et al.^20^, with the modification of the dose to 25 μg every 3 hours, and even though the high incidence of tachysystole persisted, it was not associated with a significant increase in adverse neonatal results.

With these conclusions, these same authors made a third study, attempting to find the best dose interval for minimizing the contractile problem. They compared 25 μg of misoprostol every 3 hours with a six-hour interval and concluded that the three-hour protocol was better in relation to the shorter time interval until vaginal delivery. Less oxytocin was needed and there was a lower incidence of induction failure. They also observed that tachysystole took place on average 4.4 hours after the first dose in the three-hour protocol group, suggesting the need for evaluation of this administration interval.^10^

These clinical findings are in conformity with reported time intervals for serum level maintenance for misoprostol administered by the vaginal route, in studies on its pharmacokinetics.^12^ This is exactly what was performed in this study: the evaluation of misoprostol for labor induction with a dose of 25 μg every 4 hours.

Limitations do exist in this study. It could not be carried out in a double-blind way, for obvious reasons (the application of the two methods differ), which may have caused a bias. Although physicians evaluating the patients during labor may have been aware of the patient’s group, it is unlikely that the outcomes from the study would have been affected by this. Another limitation is that the dose and administration interval were empirical, based on the experience of other authors. In spite of this, the results from this study are quite favorable towards the use of misoprostol as a modifying agent of the cervix and for inducing labor.

However, there is still a lot to learn about misoprostol and its use in obstetrics. It is not known whether lower doses or a single dose would produce the same effect. Should the first dose be vaginal (to act directly on cervix ripening) and, if necessary, the next ones be administered orally?^8^ Do ethnic and metabolic differences in populations influence the dose used?

On the basis of published studies, it needs to be decided when and under what circumstances misoprostol should be used. In cases of unripe cervices, the use of misoprostol could produce several beneficial effects, particularly a decrease in the incidence of cesarean sections.

## CONCLUSIONS

This study shows favorable results regarding the use of misoprostol as a modifying agent of the cervix and for inducing labor. Investigations need to be continued, in order to find the smallest useful dose that facilitates cervix ripening and labor induction without causing any uterine contractility alteration or harm to the fetus.
